# Local/Global contagion of viral/non-viral information: Analysis of contagion spread in online social networks

**DOI:** 10.1371/journal.pone.0230811

**Published:** 2020-04-10

**Authors:** Alon Bartal, Nava Pliskin, Oren Tsur

**Affiliations:** 1 Dept. of Pharmacological Sciences, Icahn School of Medicine at Mount Sinai, New York, NY, United States of America; 2 Dept. of Industrial Engineering and Management, Ben-Gurion University of the Negev, Beer Sheva, Israel; 3 Dept. of Software and Information Systems Engineering, Ben-Gurion University of the Negev, Beer Sheva, Israel; Central European University, HUNGARY

## Abstract

Contagion in online social networks (OSN) occurs when users are exposed to information disseminated by other users. Studies of contagion are largely devoted to the spread of *viral* information and to *local* neighbor-to-neighbor contagion. However, many contagion events can be *non-viral* in the sense of being unpopular with low reach size, or *global* in the sense of being exposed to non-adjacent neighbors. This study aims to investigate the differences between local and global contagion and the different contagion patterns of viral vs. non-viral information. We analyzed three datasets and found significant differences between the temporal spreading patterns of local contagion compared to global contagion. Based on our analysis, we can successfully predict whether a user will be infected by either a local or a global contagion. We achieve an *F*_1_-score of 0.87 for non-viral information and an *F*_1_-score of 0.84 for viral information. We propose a novel method for early detection of the viral potential of an information nugget and investigate the spreading of viral and non-viral information. In addition, we analyze both viral and non-viral contagion of a topic. Differentiating between local versus global contagion, as well as between viral versus non-viral information, provides a novel perspective and better understanding of information diffusion in OSNs.

## 1 Introduction

Contagion in an Online Social Network (OSN) is typically measured by the tendency of users to perform online activities such as re-posting or sharing of information, or adopting a new behavior after exposure to similar information or behavior respectively. For example, a user’s feed on Twitter or Facebook presents some of the online activities, e.g., posting or sharing, performed by other users whom s/he follows or is friends with. These social online platforms are designed to maximize interaction and engagement, creating an *activity network* based on a post-reply interaction, re-sharing activity, or “voting”, e.g., “like” on Facebook and “favoriting” (starring) on Twitter. Some OSNs support two types of networks, an explicit social network, and an implicit activity network. For instance, in addition to a social network of Following-Follower relationships, Twitter supports an activity network of who tweets whom [1] and a retweet (RT) activity network whose nodes are tweet authors and edges indicate paths of contagion spreading [2].

Contagion is often viewed by OSN researchers as a result of user exposure to content posted by *adjacent* neighboring users on the social graph. One way by which information can reach a user is through local exposure to posts by network neighbors whose distance from the user is 1-hop. Such exposure can lead to local information contagion in the form of infection, influence, or adoption. For example, on Twitter, exposure of a user to information can occur through her/his Follower lists [3] and can result in *local* contagion when a user retweets a message that a neighbor posted [1, 2]. However, contagion can also be triggered by non-local mechanisms [4] like content promotion [5], exposure to external sources as the mainstream media [6, 7], browsing for information [8], or recommender systems integrated into the social platform [9, 10]. This type of exposure to content, not propagated by the user’s direct neighbors, can lead to *global contagion*.

Previous studies have considered various aspects contributing to contagion. The temporal order of posted items was considered by [5, 11–14]. Structural and non-structural approaches are used to model contagion spread and, most of them [2, 15, 16] infer the spread of local contagion by users who expose network neighbors to their posted content. Moreover, most of these approaches overlook information spread by non-neighbors, assuming implicitly that information can reach a user only within the network edges. However, global contagion can result from external out-of-network events as exposure to content on mainstream media [6]. Yet, global contagion has been addressed as an aggregated phenomenon [6, 7] without studying user behavior at a micro-level. However, a better understanding of contagion spread in OSNs requires accounting for both local and global effects. In addition, most studies of contagion focus on viral content that spreads to numerous users within a short period [6, 17]. While there is no consensus on the number of contagions that make an event “viral”, it is well established that most posts are shared a scant number of times [17–19]. Yet, research on contagion spread of non-viral content is sparse [20].

Whereas most contagion studies model local contagion and spread of viral information, this study investigates local/global contagion spread of viral/ non-viral information in three datasets. To detect the depth of the reach of an information nugget, we measure the distance on the Following-relationship network from the source who originated the information, and the distance from the closest infected user to a newly infected user. We found significant differences in the spread of global versus local contagion of viral and non-viral information. Contrary to the common assumption that contagion diffuses from node-to-node, we found that contagion in OSNs also spreads globally beyond social network links. In addition, we found that on the micro-level, viral information spreads faster than non-viral information.

This work makes two main contributions to the vast body of scholarship addressing contagion spread in OSNs. First, our data analysis revealed that local contagion and global contagion are associated with significantly different temporal spreading patterns. Second, we explain the types of contagion spread by considering the time difference from the posting of the original tweet to the time of infection, the number of global and local contagions, and the different distances. Third, based on our data analysis, one can successfully detect whether a user will be infected by either a local or a global contagion with an *F*_1_-score of 0.87 for non-viral information and with an *F*_1_-score of 0.84 for viral information.

**Organization**. Section 2 sets the theoretical background for this study, followed by Section 3 which presents the methods used. Section 4 describes the different datasets, followed by the research hypotheses in Section 5. Sections 6 presents the results of the data analyses, followed by hypotheses testing in Section 7. Finally, the limitations of this study are briefly discussed and future research directions are proposed in Section 8.

## 2 Related work

### 2.1 Structural contagion models

Most contagion models focus on simple contagion and complex contagion [21, 22]. Simple contagion describes a controlled process with a contagion probability being independent of the number of exposures [22]. Complex contagion describes a process that requires multiple exposures to a contagious entity [22] and describes better than simple contagion the spread of ideas, technologies, or a behavior [22, 23]. Most information-spread studies, whether devoted to simple or complex contagion, focus on local contagion as a result of user-to-user exposure [1, 2, 24].

Addressing local contagion with structural modeling, Leskovec et al. [25] studied a recommendation graph and measured the extent to which a user’s activity in recommending a product is contagious and affects the purchase decisions of adjacent neighbors. Similarly, Sun at al. [26] studied the contagion effect on the participation of users in fan pages after some of their neighboring friends have done so, and Bakshy et al. [27] examined contagion in adopting the use of gestures among friends in the Second Life platform. The Linear Threshold Model [28] allows a user’s transition from a non-active to an active state following a similar transition in the participation of a network neighbor. Kleinberg et al. [29] used a structural transition model with a similar contagion definition of user participation and analyzed the propagation of local contagion by allowing a user to activate her/his inactive neighbors. Two-step diffusion models [15, 30], however, posit that a piece of information first spreads globally from the mainstream media to opinion leaders and, only afterward, propagates locally in a node-to-node manner from opinion leaders to a broader population.

The local neighbor-to-neighbor mechanism at the basis of the structural contagion models discussed so far, miss the global mechanisms behind exposure of OSN users to varied content by non-neighbors, beyond network structure, that can lead to global contagion [4, 6]. For example, a contagion event like retweeting (RT) a message on Twitter by re-posting someone else’s tweet [1, 3] and passing interesting pieces of information to followers, is limited neither to viral information that a user is exposed to nor to a local user-to-user mechanism only. Since contagion can occur beyond pairwise interaction, through higher-order structures than neighbor-to-neighbor [4, 6], it is important to consider contagion by all network users upon modeling contagion.

Detecting both local and global contagion mechanisms while considering network structure is crucial to better understanding human behavior online, as manifested by user interactions [31]. In the case of Twitter, users are exposed to information posted by non-neighbors via exposure to hashtags [32] as well as to promoted content on a user’s Timeline feed [13], which includes tweets of accounts that a user follows as well as content tweeted by non-neighbors either by advertisers purchase or tweets ranked as having a large engagement potential [33]. Similarly, Facebook and Reddit allow global exposure via trending topics that appear on a user’s front page [34]. This facilitation of global exposure in OSNs raises the need to consider non-structural contagion modeling, beyond network structure, as discussed next.

### 2.2 Non-structural contagion models

Non-structural contagion models (e.g. [35]) do not rely on the structure of the network to infer contagion. The Susceptible-Infected-Resistant (SIR) model [36] and the Susceptible-Infectious-Susceptible (SIS) model [36], for example, assume that every individual has the same probability to be infected. In SIR and SIS models, all users thus have the same contact rate, which is indicated by an edge formation in a network. However, contagion in OSNs is not evenly distributed among users [37] and is likely to depend on exposure rates [6], as modeled by the Linear Influence Model [6], which assumes a static network structure in which the infection likelihood of a user is affected by the number of contagious network users [38].

Aiming to better explain contagion spread with non-structural modeling, Wang et al. [39] modeled contagion by mainly focusing on temporal and topological dynamics while integrating a single topological feature that represents the distance between the infecting and the infected users. Global contagion can also result from homophily-driven diffusion by a peer-to-peer influence model [39], where the user interface has a substantial effect on the contagion process [1]. Other studies [12, 40] focus on the linguistic properties of textual information in predicting contagion spread. In recent years, evidence of global contagion due to exposure to external sources like mainstream media was found by [6, 7, 11] among others.

Since this study aims to investigate not only local versus global contagion but also the spread of viral and non-viral information, structural contagion models have been reviewed in the previous sub-section and non-structural contagion models have been reviewed in the present sub-section. Another goal of this study is to go beyond most studies [18, 20, 41–47] which, as discussed next, focus on viral contagion, largely ignoring contagion spread of non-viral information.

### 2.3 Viral versus non-viral contagion

Analyzing viral contagion, Romero et al. [2] modeled complex contagion of the 500 most frequent hashtags in a Twitter dataset of three billion messages with a median hashtag count of over 93,000 occurrences. Dow et al., [17] studied the spread of 1-million images among Facebook users, restricting their analysis to viral images shared at least 100 times. The “Yes We Can” slogan used in the 2008 U.S. elections reached over 20 million views, demonstrating yet another analysis of viral contagion [48]. The number of contagion events that make a piece of information viral differs among studies. Gleeson et al. [14] found that the structure of the network and temporal dynamics can explain sub-critical (non-viral) cascades.

One of the lowest contagion events was defined by Myers et al. [6] who restricted their analysis to messages shared at least 50 times, while Dow et al. [17] restricted their viral-contagion analysis to images shared at least 100 times. Liben-Nowell et al. [42] measured cascades with hundreds of steps, showing that re-sharing contagion activities have the potential of information spreading to millions of users [20].

The reach distribution of contagious information is long-tailed since most information nuggets are shared only a small number of times if shared at all [18, 49]. Cheng et al. [18], who found that the cascade size distribution of photos posted by users follow a power-law curve with an exponent of *α* = 2.2, concluded that most information nuggets are scarcely shared further. It is reasonable, therefore, to assume that there exists a contagion mechanism in the few cases in which an information nugget is shared enough times to create a viral contagion.

Several studies [41, 45] have addressed the important task of detecting whether a piece of information will go viral [20] by focusing on information cascades as well as by modeling the contagion spread of hashtags [43], behavioral dynamics [46] or, for example, YouTube views [50]. Other studies tried to predict the size of information cascades by using network features [18, 47]. For instance, Cui et al. [44] applied a logistic model that considers the relative importance of each node given the list of previously infected nodes. Most of these approaches, however, lack the ability to detect virality early.

The scarcity of research regarding contagion spread of non-viral content and regarding global effects on the contagion mechanism has motivated us to distinguish between local versus global contagion and viral versus non-viral information. Complementing and contributing to the body of OSN scholarship about contagion spread of information, we focus on non-viral contagion as a mean to better understand both local and global contagion mechanisms and the nature of human behavior online.

## 3 Methodology

We begin by defining viral and non-viral information nuggets in Section 3.1. Then, in Section 3.2, we outline the methodology developed for detecting local and global contagion spread of viral and non-viral information nugget in the form of a particular message. Next, in Section 3.3, we generalize the methodology of contagion spread of a particular message to the contagion spread of a topic by defining local and global contagion of viral and non-viral topics. Finally, In Section 3.4, we present an innovative approach for detecting the virality of a tweet in its early stages.

### 3.1 Defining information virality

According to Dow et al. [17], viral events are manifested by resharing of photos on Facebook at least 100 times. According to Goel et al. [15], rare events are those manifested by resharing tweets on Twitter—0.025% of viral events identified by diffusion trees with at least 100 nodes. Following these authors, we define an information nugget as viral if it is shared at least 100 times and as non-viral if it is shared 10 to 99 times, overlooking information nuggets shared less than 10 times since their contagion spread signature is too low to allow studying contagion spread.

### 3.2 Detecting contagion spread of information

Consider a directed social network *G* = (*V*, *E*) as defined in Table 1. A post of User *v*_*j*_ ∈ *V* can result in local contagion of User *v*_*i*_ ∈ *V* who Follows *v*_*j*_, starting a cascade of local contagions. Since contagion is time-dependent, we define it more formally while considering the temporal activities of users.

**Table 1:** A summary of variables definitions.

Parameter	Particular (original) message	Topic
Contagion	Users are infected by a single message *w*	Users are infected by a topic (*p*) that can contain a set (*W*) of particular messages: {*w*_1*p*_, …, *w*_*np*_}∈*W*
Social network *G* = (*V*, *E*)	*V* ≔ Users *E* ≔ Following relationships among users *v*_*j*_ → *v*_*i*_: *v*_*j*_ exposed *v*_*i*_	*V* ≔ Users *E* ≔ Following relationships among users *v*_*j*_ → *v*_*i*_: *v*_*j*_ exposed *v*_*i*_
Activity network	*G*_*Tw*_ = (*V*_*Tw*_, *E*_*Tw*_) *V*_*Tw*_ ≔ Users who shared/ posted *w* at times *t*_*i*_ ≤ *t*_*k*_ *E*_*Tw*_ ≔ Resharing activities *v*_*i*_ → *v*_*j*_: *v*_*i*_ was infected by *v*_*j*_	*G*_*TW*_ = (*V*_*TW*_, *E*_*TW*_) *V*_*TW*_ ≔ users who shared/ posted any *w*_*μp*_ ∈ *W* at times *t*_*i*_ ≤ *t*_*k*_ *E*_*TW*_ ≔ resharing activities *v*_*i*_ → *v*_*j*_: *v*_*i*_ was infected by *v*_*j*_
Local contagion of User *v*_*i*_	*e*_*ji*_ ∈ *E* and *e*_*ij*_ ∈ *E*_*Tw*_	*e*_*ji*_ ∈ *E* and *e*_*ij*_ ∈ *E*_*TW*_
Global contagion of User *v*_*i*_	*e*_*ji*_ ∉ *E* and *e*_*ij*_ ∈ *E*_*Tw*_	*e*_*ji*_ ∉ *E* and *e*_*ij*_ ∈ *E*_*TW*_
Viral event	∑*share*(*w*) ≥ 100	∑_*w*_*μp*_ ∈ *W*_ *share*(*w*_*μp*_) ≥ 100
Non-viral event	10 ≤ ∑*share*(*w*) ≤ 99	10 ≤ ∑_*w*_*μp*_ ∈ *W*_ *share*(*w*_*μp*_) ≤ 99
Analyzed dataset	DS1, DS2	DS3
*d* ≔ *d*(*v*_0_, *v*_*i*_)	Distance on *G* from the source who originated *w* to an infected User *v*_*i*_	Distance on *G* from the source who originated *w*_*μp*_ ∈ *W* to an infected User *v*_*i*_
*d*_*ca*_ ≔ *d*(*v*_*j*_, *v*_*i*_)	Distance on *G* from any adopting user or the user who originated *w* to an infected User *v*_*i*_.	Distance on *G* from any adopting user or the user who originated any *w*_*μp*_ ∈ *W* to an infected User *v*_*i*_.

*t*_*k*_—time of infection of node *v*_*i*_; *v*_*j*_—infecting node (who adopted the information); *v*_0_—the user who originated the information.

Let *w* denote an information nugget, like an original tweet, posted at Time *t*_0_ by User *v*_0_ ∈ *V*. Contagion spread, like retweeting, of *w* at times *t*_1_…*t*_*k*_ by users *v*_1_, …, *v*_*j*_ ∈ *V*, along with *v*_0_, could be thought of as a temporal *activity network* *G*_*Tw*_ = (*V*_*Tw*_, *E*_*Tw*_) as defined in Table 1. The social network *G* and the activity network *G*_*Tw*_ allow us to define next, as summarized in Table 1, local and global contagion events.

#### Local contagion event

A contagion event of User *v*_*i*_ is local if *e*_*ji*_ ∈ *E* and *e*_*ij*_ ∈ *E*_*Tw*_. In other words, *v*_*i*_ has shared an original post *w* after one of the users s/he follows has shared or posted *w*.

#### Global contagion event

A contagion event of User *v*_*i*_ is global if *e*_*ji*_ ∉ *E* and *e*_*ij*_ ∈ *E*_*Tw*_. In other words, *v*_*i*_ has shared a post *w* before any of the users s/he follows has shared or posted *w*.

**Fig 1:**
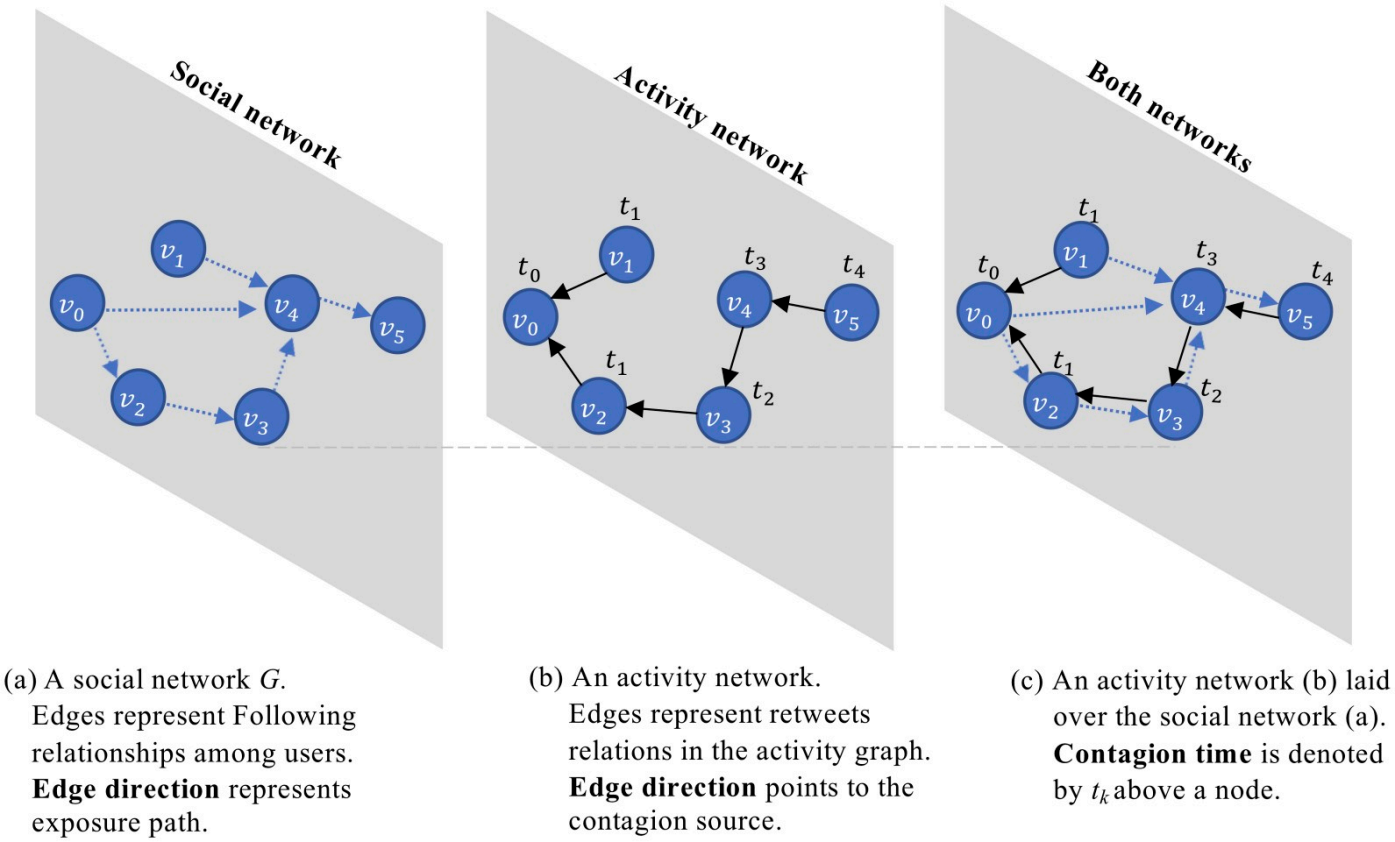
An activity network with a social network. Distances are measured on the social network.

Fig 1 illustrates an example of local and global contagion spread. Consider a network (Fig 1) in which User *v*_0_ posts a tweet *w* at Time *t*_0_, exposing Users *v*_2_ and *v*_4_ who Follow her/him. At Time *t*_1_, *v*_2_ retweeted *w*, demonstrating local contagion since s/he follows *v*_0_, while *v*_1_ retweeted *w*, demonstrating global contagion since s/he does not follow *v*_0_. The retweets of *v*_3_ at *t*_2_, *v*_4_ at *t*_3_, and *v*_5_ at *t*_4_ all demonstrate local contagion events since their opposite edges in *E*_*Tw*_ exist in *E*.

To detect the reach depth of an information nugget *w*, the distance *d*, is defined (1):

1. *d*—The distance on *G* from the source who originated *w* to a newly infected user.

To detect whether a contagion is local/global, distance *d*_*ca*_ is defined in (2).

2. *d*_*ca*_—The distance on *G* to a newly infected user from the closest infected user who adopted *w* or from the user who originated *w*.

The definitions of the variables are summarized in Table 1.

Returning to Fig 1, consider an example where the retweeting source User *v*_0_, and her Follower *v*_3_ are the only members of *V*_*Tw*_ at Time *t*_*k*−1_. Next, global contagion spreads to User *v*_5_ at Time *t*_*k*_. The minimal distance from any user in *V*_*Tw*_ to User *v*_5_ before *t*_*k*_ is *d*_*ca*_(*v*_3_, *v*_5_) = 2, or *d*_*ca*_(*v*_0_, *v*_5_) = 2.

Several posts that describe the same subject can be grouped into a topic, and analyzing the contagion spread of a topic instead of the spread of a more specific nugget can uncover more realistic patterns of contagion spread [51].

### 3.3 Detecting contagion spread of a topic

A topic contagion event occurs when a single User *v*_*i*_ posts several original messages about the same subject, with each original message potentially shared by different users.

For example, assume that User *v*_0_ (Fig 1) tweeted twice about the same specific topic—the discovery of the Higgs boson particle—described further in Section 4. The First tweet (i) was: #CERN scientists inexplicably present #Higgs #boson findings, and the second tweet (ii) was: New result from #LHC reinforces belief that the particle has been found. User *v*_2_ who follows *v*_0_, retweeted (i) exposing to the topic a follower—User *v*_3_—who does not follow *v*_0_. Next, *v*_3_ retweeted (ii). This contagion sequence facilities an event of local topic contagion: *v*_3_ was exposed to a topic and to *v*_0_, upon being infected by *v*_2_ whom s/he follows.

Our definitions of viral topic contagion and non-viral topic contagion are similar to the definitions of particular viral and non-viral tweets suggested in Section 3.2. A topic is viral if its set of original messages were shared at least 100 times and is non-viral if the set of its original messages were shared 10 to 99 times. There are two differences between contagion of a particular tweet and contagion of a topic. First, the number of retweets of each original message within a topic that was posted by the same User *v*_*i*_ are summed. Second, while users who share a particular message whose origin is User *v*_*i*_ are considered infected, a user infected by a topic is one who shares any of *v*_*i*_’s original messages on the same topic. Stated differently, in the case of topic contagion, a user can be directly exposed to one message whose origin is *v*_*i*_ but share another message on the same topic whose origin is the same user *v*_*i*_.

Topical contagion is a more realistic scenario as users are infected by a concept rather than by a fixed sequence of characters.

For a more formal definition of topic contagion, the social network *G* is defined as suggested in Section 3.2, and a topical contagion is then defined: A set of original tweets *w*_1*p*_, …, *w*_*np*_, posted within a selected time interval by User *v*_0_ ∈ *V* about a topic *p* (denoted by *w*_*μp*_, *μ* = 1, …, *n*). The retweeting spread of *w*_*μp*_ ∈ *W* at times *t*_1_…*t*_*k*_ by Users *v*_1_, …, *v*_*j*_ ∈ *V*, along with User *v*_0_ who originated *w*_*μp*_, form a temporal activity network *G*_*TW*_, which is laid over *G*. In *G*_*TW*_, Nodes *V*_*TW*_ depict *v*_0_ as well as users who retweet any *w*_*μp*_ ∈ *W* at Times *t*_*i*_ ≤ *t*_*k*_ and Edges *E*_*TW*_ represent retweet relations. These definitions are also summarized in Table 1.

#### Local topic contagion event

A topic contagion event of User *v*_*i*_ is local if *e*_*ji*_ ∈ *E* and *e*_*ij*_ ∈ *E*_*TW*_. In other words, *v*_*i*_ has shared an original post *w*_*μp*_ ∈ *W* *after* one of the users s/he follows has shared or posted *w*_*μp*_ ∈ *W*.

#### Global topic contagion event

A topic contagion event of User *v*_*i*_ is global if *e*_*ji*_ ∉ *E* and *e*_*ij*_ ∈ *E*_*TW*_. In other words, *w*_*μp*_ ∈ *W* was not posted by any user that User *v*_*i*_ follows, and *v*_*i*_ shared *w*_*μp*_ ∈ *W* *before* any of the users s/he follows has shared or posted *w*_*μp*_.

We define the two types of distances (*d* and *d*_*ca*_) similar to the manner defined for particular tweets in Section 3.2, as summarized in Table 1.

### 3.4 Detecting tweet virality in early stages

Our goal is to detect whether a tweet will become viral at its early stages—before it becomes viral. To achieve this goal, we developed the Back-in-time (BIT) approach that allows us to study the spreading patterns of viral tweets before they became viral and compare them to the spreading patterns of non-viral tweets. To analyze contagion spread of viral information in its early stages before it became viral, we roll a viral tweet back in time to a point when it was still a non-viral tweet (i.e., having 10 to 99 retweets). At the end of this stage, both rolled-back tweets (BIT-tweets) and non-viral tweets have 10 to 99 retweets. Aiming to show that the spreading patterns of BIT-tweets and non-viral tweets differ significantly, we compare the spreading patterns of BIT-tweets with the spreading patterns of non-viral tweets. Under the BIT approach, a viral tweet is sent back in time and the number of retweets (retweet-count) it had back then is determined, via three steps: (i) Learn the retweet count distribution of non-viral tweets by creating a kernel density estimate (KDE) of their retweet-counts. Accounting for non-viral tweets, the left-most and right-most points of the grid at which the density is estimated are 10 and 99 respectively; (ii) Randomly sample from the KDE a number *y* that represents a retweet-count and assign it to an original viral tweet. For that purpose, we draw a point *x*_*i*_ from the set of points *x*_1_, …, *x*_*n*_ included in the KDE. Then, we draw a value *y* associated with *x*_*i*_ to ensure that more probable values of non-viral retweet-counts in the distribution are more likely to be randomly sampled; (iii) Roll a viral tweet back in time by keeping the oldest retweets, such that its retweet-count is equal to *y*.

Next, we describe the three datasets used in this study.

## 4 Datasets description

**Dataset 1 (DS1)** contains local/global contagion as well as viral/non-viral messages and was collected using Twitter stream API, by following four Steps. (i) Hebrew tweets were collected during five months prior to the Israeli elections of April 2019, since their politics content can potentially go viral and allow us to select, in the next step, a set of active users who posted at least once; (ii) A set *C* of several hundreds of active users, containing politicians and journalists, was defined; (iii) All tweets that mention, or interact with each *u*_*i*_ ∈ *C* from December 2018 to January 2019 were captured, where each original tweet has the potential to be contagious and initiate a series of contagion events; and (iv) Collect followers of users *u*_*i*_ ∈ *C*, who posted an original tweet.

**Dataset 2 (DS2)** facilitates studying contagion spread of non-viral information by using the Twitter stream API to first collect into the *initial* dataset, during March 2017, all the accounts of users who posted public tweets that mention the president of the United States (US) or a member of the US Congress. Then, the appropriate dataset was curated in five steps: (i) Users who posted a tweet in the initial dataset were included in DS2 if the user’s Followers list and Friends list range between 100—to avoid inactive users—and 1,000—to avoid celebrities who tend to go viral; (ii) The 200 most recent tweets of each user selected in Step *i* were collected (as done in [52]), without topic limitation, thus countering the possible bias toward topics about politics in the initial dataset. The most recent tweets were collected since the older a tweet, the more likely that it is that its’ retweet list will be missing a user and possibly bias the results; (iii) To include non-viral tweets only, each tweet with 10 to 100 retweets (RTs), collected in Step *ii* was kept; (iv) Accounts of users who retweeted an original tweet in Step *iii* were kept in DS2; and (v) The Followers-list of users kept in Step *iv* were collected.

To verify that the retweet counts of non-viral tweets did not grow to be viral, Non-viral tweets in DS1 and DS2 were queried using Twitter API at least a month after their posting date and only non-viral tweets were considered.

**Dataset 3 (DS3)** was curated from the dataset, described in [53], to study the way topical contagion spreads within a time interval, covering tweets posted from July 1^*st*^ to July 7^*th*^, 2012 about the discovery of the Higgs boson subatomic particle. DS3 contains interactions on the same topic by mentions, replies, retweets, and original tweets with at least one of the following keywords or hashtags: “lhc”, “cern”, “boson”, “higgs”. DS3, curated in three steps Included: (i) Users who posted an original tweet; (ii) The RT list for each original tweet; and (iii) The Following relationships among users, selected in the two earlier steps.

DS3 differs from DS1 and DS2 in containing data about contagion spread of a topic instead of data about the spread of a single original tweet. Homophily often explains social relationships [54]. Since viral and non-viral tweets in DS3 are discussing the same topic, users who retweeted them might be socially connected by a Following relationship due to similar topic interests. Therefore, our analyses are based on the combined social network formed by users in DS3 who retweeted or posted viral and non-viral content.

Table 2 summarizes the three analyzed datasets after preprocessing.

In the context of the background and the proposed differentiation approach, we address four hypotheses next.

**Table 2:** A summary of the analyzed datasets.

Dataset	Information type	# Contagion events	|*V*_*Tw*_|	|*E*_*Tw*_|	|*V*|	|*E*|	Global	Local
DS1	Non-viral	1,950	4.1k	26.6k	0.85M	3.21M	12.14%	87.86%
DS1	Viral	127	42.2k	132.1k	10.3M	53.3M	21.75%	78.25%
DS2	Non-viral	3,156	25.6k	172.2k	11.04M	141.4M	18.29%	81.71%
DS3	Non-viral	3,359	67.1k	90.2k	0.256M	7.7M	12.8%	87.2%
DS3	Viral	556	139.3k	299.3k	0.256M	7.7M	16.9%	83.1%

k—thousands; M—millions; Global—% global contagions; Local—% local contagions.

## 5 Research hypotheses

Global contagion is not limited to a node-to-node spreading mechanism over time [4] and, hence, we hypothesize:

***H1***. *Local and global contagion have different temporal spreading patterns in an OSN*.

Only a small fraction of information goes viral and reaches a large audience. Detecting in the early stage of its life-cycle whether the information would evolve to be viral or not is extremely useful [17]. Due to the difference between viral and non-viral information, expressed by the temporal dynamics of information spread, we hypothesize:

***H2***. *Viral and non-viral information have different local and global contagion spreading patterns, starting at an early stage of the appearance of the information*.

A piece of information can spread by a single sharing activity such as a retweet. Several messages on the same subject can be grouped by a topic [2, 55]. Analyzing contagion spread of such a topic can uncover realistic patterns of contagion spread. Hence, we hypothesize:

***H3***. *Topic contagion has similar temporal spreading patterns to contagion spread of information*.

## 6 Results of data analyses

### 6.1 Analysis of contagion reach depth

This section describes how we detect a contagion and measure its reach. Given a network, local contagion begins at some node and then spreads over the edges. Typically, local contagions are measured on the activity network [56]. However, the social network plays a fundamental role in the dynamics of spreading [53, 57, 58].

We detect a contagion using the activity network (*G*_*Tw*_) and measure the depth it reaches on the social network (*G*). The depth is defined as the largest distance (*d*) that the information (*w*) spreads from the user originating the information (Table 1).

To detect the reach depth of an original tweet (*w*), each user retweeting *w* was assigned a distance (*d*) on *G* and in the absence of a path on *G*, an infinite distance (*INF*) was assigned. Instead of INF, a path might exist at larger distances by collecting wider circles of Following-lists but contagion at such larger distances is global and, thus, does not affect interpreting the results.

Most contagion events of both viral and non-viral information are local (*d* = 1). Viral information (Fig 3a) has a contagion reach depth *d* ≤ 18, whereas non-viral information (Fig 3b), has a shorter contagion reach with depth *d* ≤ 7.

For both viral information (when *d* ≤ 9, *d* ∈ [13, 18]), and non-viral information (when *d* ≤ 7) in DS1, if a path exists on *G*, the more distant a user is from the source who originated the information (User *v*_0_), the less likely s/he is to retweet *w*. We observed a trend shift for viral information when *d* ∈ [10, 12], and found that the more distant a user is from the source User *v*_0_, the more likely s/he is to retweet *w*.

For non-viral information, DS2 presents similar trends to DS1 (Table 3) for *d* ≤ 9, *d* ≠ 8 (Fig 3c), if a path exists on *G*, the more distant a user is from the source User *v*_0_, the less likely s/he is to retweet *w*. For *d* = 8, users are more likely to be infected than *d* = 7. This finding might be attributed to global contagion (e.g. mass media or the Timeline). Similar to DS1, most contagion events in DS2 are local (*d* = 1).

**Table 3:** Contagion spread: The larger the distance the less likely a user will retweet.

	Non-viral			Viral		
Distance	DS1	DS2	DS3	DS1	DS2	DS3
*d*	*d* ≤ 7	*d* ≤ 9, *d* ≠ 8	*d* ≤ 13	*d*_*ca*_ ≤ 8	–	*d* ≤ 17, *d* ≠ 10
*d*_*ca*_	*d*_*ca*_ ≤ 7	*d*_*ca*_ ≤ 6	*d* ≤ 7	*d*_*ca*_ ≤ 8	–	*d*_*ca*_ ≤ 9
Figure	Fig 3b	Fig 3c and 3d	Fig 3f	Fig 3a	–	Fig 3e

*d* and *d*_*ca*_—are defined in Table 1.

To better understand contagion spread of viral and non-viral information, we analyze next, contagion spread by type.

### 6.2 Analysis of contagion types

**Fig 2:**
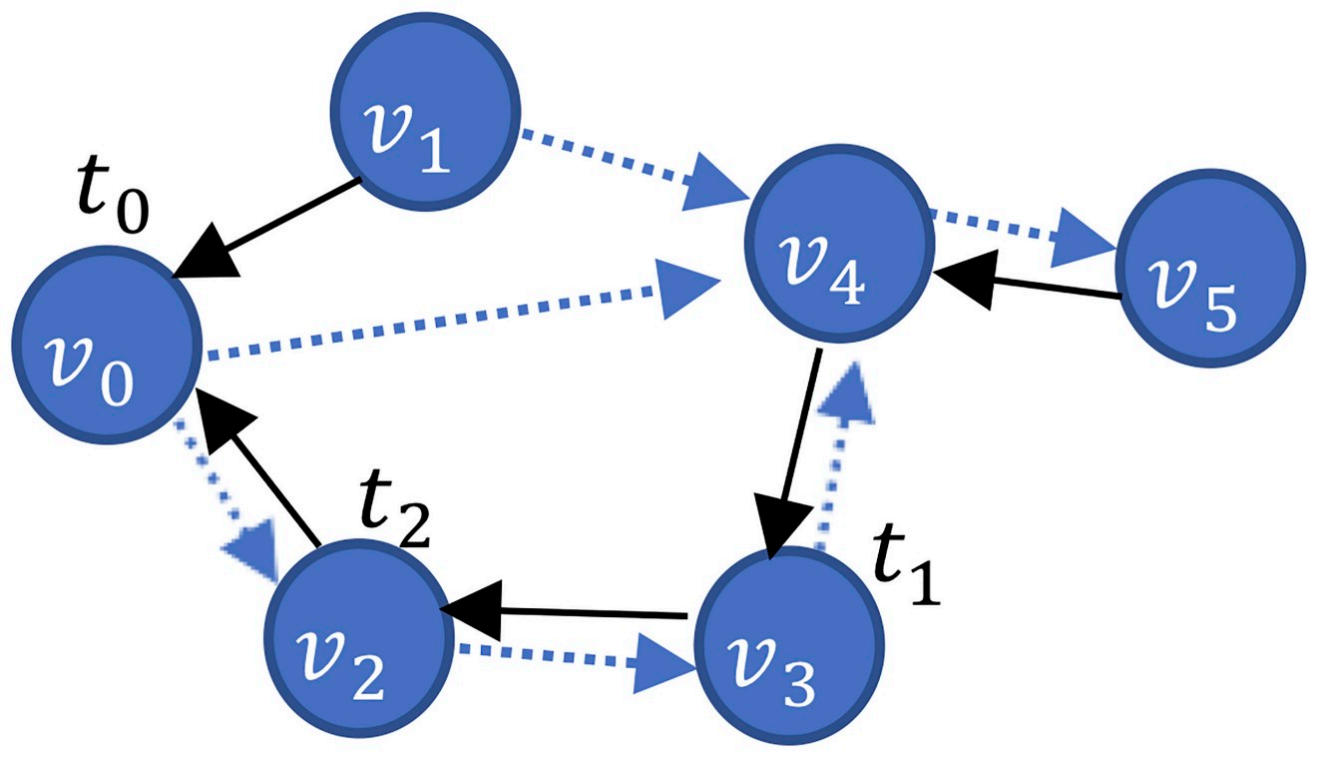
An activity network with a social network illustrating global contagion.

This section explains how we detect local and global contagion. As explained in Section 3.2 and demonstrated in Section 6.1, local contagion results when *d* = 1. However, the mechanism for global contagion is different. Fig 2 demonstrates global contagion where User *v*_0_ is the source of *w* and User *v*_3_ retweets *w* at Time *t*_1_ before User *v*_2_ retweets *w* at Time *t*_2_. This order of events implies global contagion of User *v*_3_.

**Fig 3:**
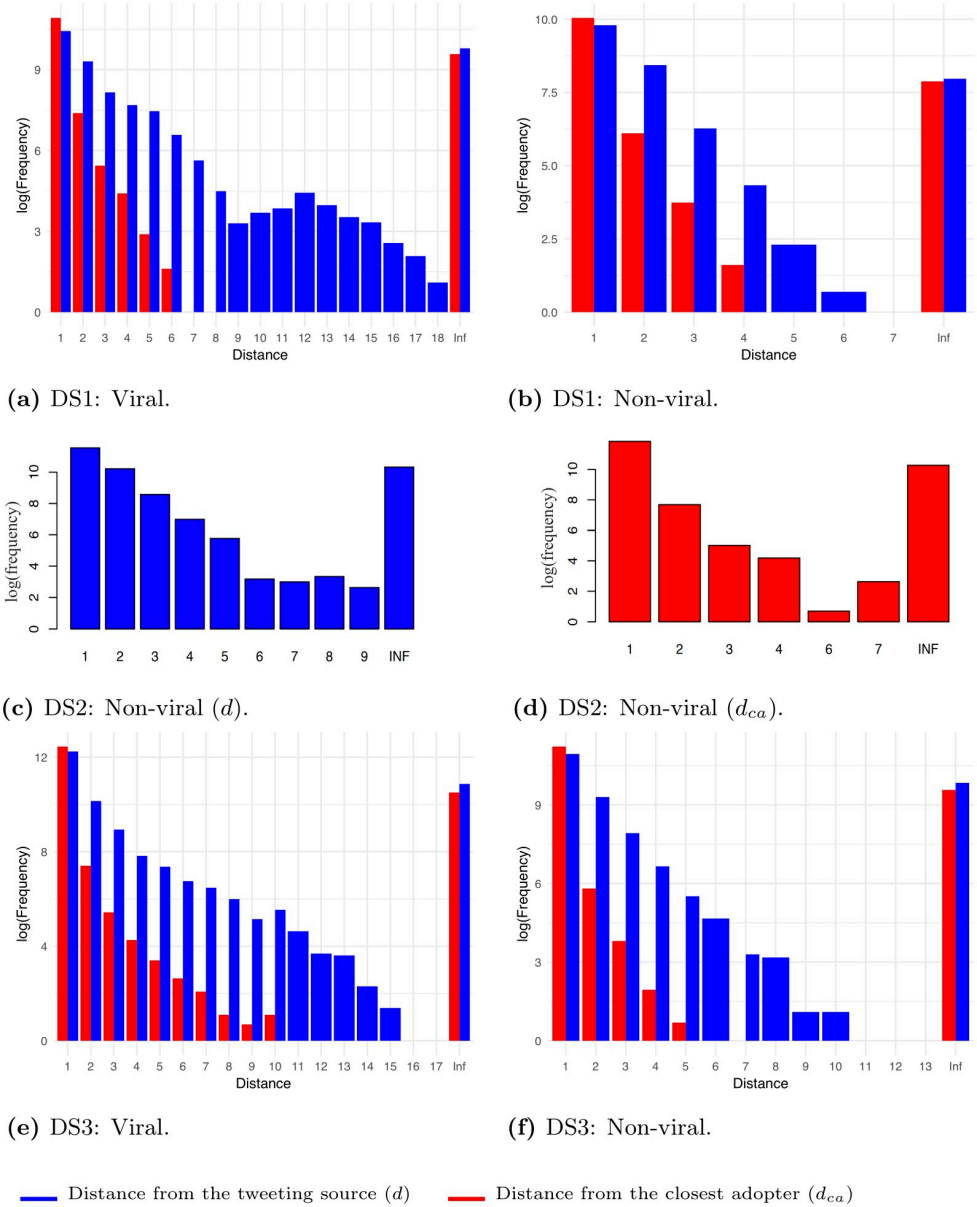
Distributions of distances, measured on the social network (G) for each of the three analyzed datasets, DS1 to DS3 (Table 2).

To account for such cases, we assigned to each infected node a local or global contagion type as described in Section 3.2. Table 2 shows the percentage of local and global contagions in each dataset. Next, we computed *d*_*ca*_ (Table 1). Analyzing DS1 for viral information, Fig 3a depicts two types of distances and, by analyzing DS1 for non-viral information Fig 3b also presents two types of distances. By analyzing DS2, which contains non-viral information only, Fig 3c and 3d depict two types of distances as well. Similarly, by analyzing DS3 for viral topic spread, Fig 3e depicts two types of distances and, by analyzing DS3 for non-viral topic spread, Fig 3f depicts two types of distances.

In Fig 3, which presents a distribution of *d* and *d*_*ca*_ distances as measured on the social network *G* for each of the analyzed datasets D1 to D3, *d*_*ca*_ = 1 indicates the existence of a Following relationship among the closest adopter and an infected user, thus demonstrating local contagion. The most frequent contagion mechanism is depicted by the highest bar. On the other hand, *d*_*ca*_ > 1 indicates no Following relationship among the closest adopter and an infected user, thus demonstrating global contagion.

Table 3 summarizes these indications. In both DS1 and DS2, local contagion is the most frequent contagion mechanism (*d*_*ca*_ = 1), and the closer a user is to an adopter, the more likely s/he is to retweet. It is likely that a user who is retweeting at *d*_*ca*_ < 8 was exposed to content via network neighbors by manually crawling through the feeds of neighbors and neighbors of neighbors, or through exposure to the Twitter Timeline that presents tweets of accounts that a user follows. These results are in line with findings of previous studies on neighbor-to-neighbor contagion [1, 2, 24] that network members have a significant influence on their neighbors.

For global contagion spread of viral and non-viral information, in both DS1 and DS2, the larger the distance the less likely a user is to retweet *w*. In DS1 for viral information when *d*_*ca*_ > 8 (Fig 3a) and for non-viral information when *d*_*ca*_ > 7 (Fig 3b), as well as in DS2 when *d*_*ca*_ > 6 (Fig 3d): the larger the distance, the more likely is a user to retweet *w*. These finding can be attributed to the global contagion, resulting from global exposure to external sources. Since non-viral tweets are less likely to be covered by the mainstream media yet their global contagion distances (Fig 3b and 3d) are similar to those of viral tweets (Fig 3a), it is reasonable to conclude that the Twitter Timeline algorithm exposed users to non-viral information. Due to the low available time and limited attention of users [51], information pushed on one’s Timeline significantly increases the contagion rate [1, 19, 33]. Thus, it is more likely that the global contagion observed in Fig 3b and 3d occurred due to content promoted by the Timeline algorithm rather than due to local neighbors.

Each added local contagion expands the circles of exposed users. Hence, the number of globally exposed users at an arbitrary distance of *d*_*ca*_ also increases and might lead to global contagion. Aiming to learn the effect of each added local contagion on global contagion spread, we examine next the explanatory power of local contagion on the number of global contagions.

### 6.3 Explaining contagion spread

Our goal is to explain contagion spread by type, whether local or global while differentiating between viral and non-viral information. We developed one model for viral information and another model for non-viral information. Given contagion events, we aim to predict whether a user will be infected by a local or by a global contagion.

We measured the following explanatory terms for each individual user at the time of adoption, when s/he retweeted the information.

1. Δ*T*_*o*_—Time difference from the posting of the original tweet.
  Captures bursty user interactions [59, 60], which can explain contagion spread [61].
2. *GC*—Total number of global contagions.
  Captures contagion spread similar to non-structural models (Section 2.2).
3. *d*—Distance from the tweeting source (Table 1).
  Accounts for our findings about the likelihood of a user to retweet (Fig 3a, 3b and 3c). Note, a local contagion can occur even when *d* > 1.

Table 4 presents the results of a logistic regression model fit with least squares, separated by tweet virality, where the outcome variable is local or global contagion, and local contagion is the base parameter. The Δ*T*_*o*_ variable yields the greatest explanatory power on the contagion type of users for both viral, and non-viral information.

**Table 4:** Logistic regression results.

Term	Non-viral	Viral
*Intercept*	-3.45**	-3.30**
Δ*T*_*o*_ × 10^−3^	-9.48*	12.83**
*GC* × 10^−4^	-38.60**	-3.31**
*d* × 10^−4^	6.38**	5.09**
*F*_1_ − *score*	0.87	0.84

** *p*_*value*_ < 2*e* × 10^16^,

*p_*value*_ < 0.01

**Non-viral information**. A significant negative coefficient of Δ*T*_*o*_ indicates that the larger the time difference from the posting of an original tweet, the less likely a user to be infected via global contagion and, thus, local contagion is more likely.

**Viral information**. The opposite is found: the larger Δ*T*_*o*_, the more likely a user to be infected by a global contagion. Viral information is persistent in the sense that repeated exposures to information are more likely to have contagion effects [2].

For both models, a significant negative coefficient of the term *GC* indicates that the more global contagions occur in a network, the less likely another global contagion will occur. This finding can be attributed to one’s reluctance to retweet information of whom s/he does not know [62]. A single global contagion, e.g. of a user within a clique, can start a cascade of local contagions. Thus, local contagion minimizes the chance of a global contagion by minimizing the number of non-adopters if it spreads faster than global contagion. We test this assumption in Section 6.4. Finally, a significant positive coefficient of the *d* Term in both models indicates that the larger the distance from the user originating the tweet, the higher the probability of global contagion. To estimate model performance, we used for training in the viral group and the non-viral group 70% of the data and for testing 30%. The performance of each model is consolidated into the F_1_-score measure. Since an F_1_-score of 1.0 means perfect precision and recall, an F_1_-score close to 1.0 in Table 4 implies better model performance.

Since time difference has the largest explanatory power (Table 4), we present next, a temporal analysis of contagion spread of information.

### 6.4 Temporal analysis of contagion spread

**Fig 4:**
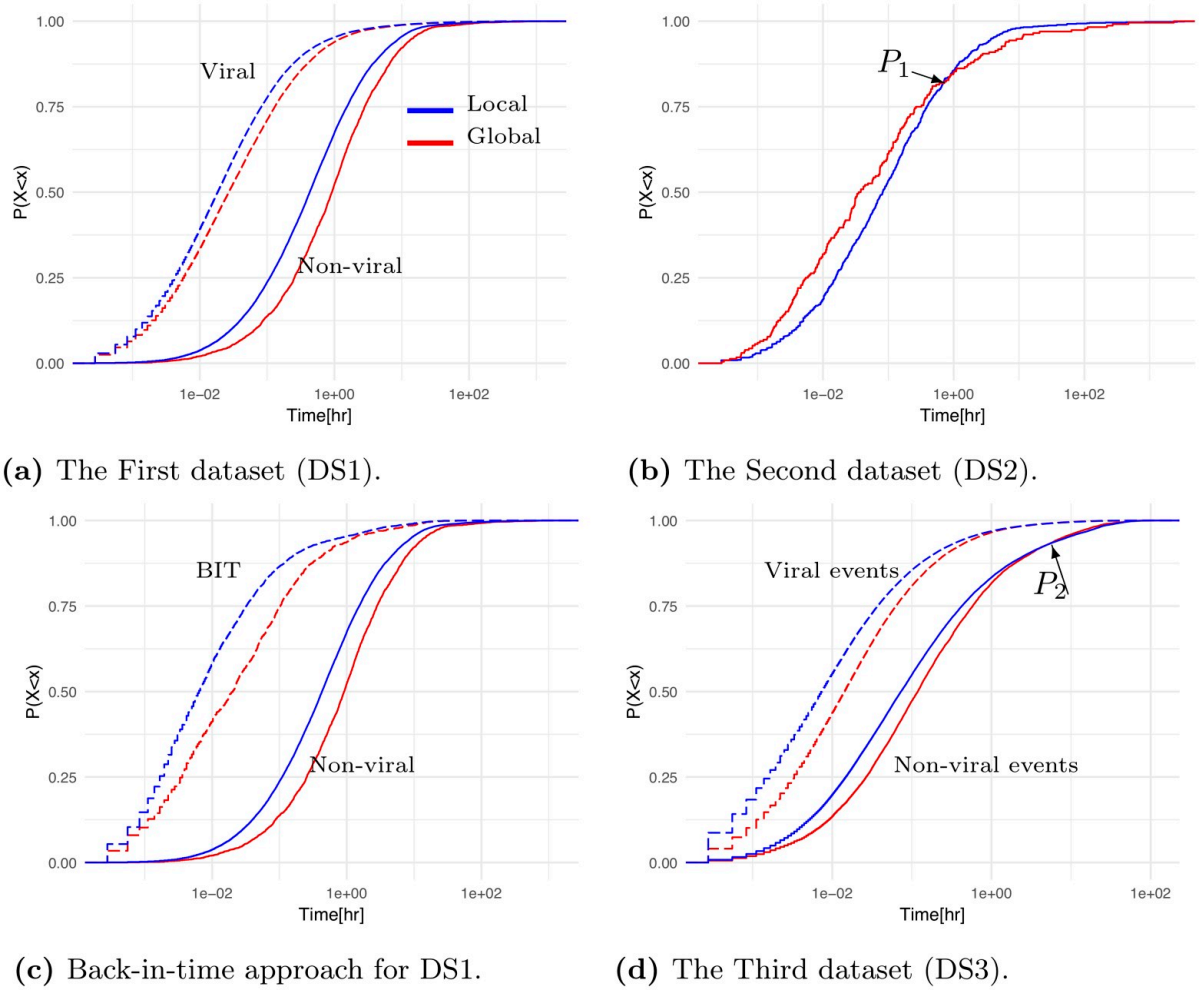
ECDFs of inter-retweet times. The time axis scale is transformed into a log scale.

To analyze the patterns of contagion spread, we calculate the time difference between every two consecutive retweets, i.e. inter-retweet times of viral and non-viral tweets. For DS1, Fig 4a presents four empirical cumulative distribution functions (ECDFs) of inter-retweet times that correspond to each contagion type, local versus global, and virality: viral tweets versus non-viral tweets.

For DS1, the two ECDF curves (Fig 4a) for local and global contagion of viral tweets are located above the two ECDFs of non-viral tweets, showing that users are more likely to retweet viral information. Regardless the of tweet vitality, the local contagion curve is located above the global contagion curve (Fig 4a), showing for DS1, that a user is more likely to retweet information tweeted or retweeted by users whom s/he follows. Unlike DS1, however, the ECDF of global contagion for DS2 (Fig 4b) is located above the ECDF of the local contagion up to Point *P*_1_, indicating that in DS2 local contagion spreads more slowly than global contagion. DS2 users retweeting due to global contagion, respond quicker than users infected by local contagion. At *P*_1_, 88% of the inter-retweet times of users were under 2 hours and afterward, the spread of global contagion is slightly slower than the spread of local contagion.

Since DS2 contains non-viral information, which is less likely covered by mainstream media, the Twitter Timeline algorithm likely promoted information up to Point *P*_1_. Since the algorithm constantly changes [33] and affects the spread of global contagion, however, the algorithm likely stopped promoting the information at Point *P*_1_.

Our findings show that Twitter users interact in bursts in the sense that short periods during which they send several tweets are separated by long periods of reduced activity [60, 63]. For DS1, the results show that local contagion spreads faster than global contagion regardless of tweet virality. Furthermore, regardless of it is local or global, contagion of non-viral information spreads faster in DS2 than in DS1 (Table 5).

**Table 5:** Time when 80% of inter-retweet times of local and global contagion are reached, considering non-viral information.

Dataset	Global contagion (80%)	Local contagion (80%)
*DS*1	3.6 hours	1.8 hours
*DS*2	0.44 hours	0.91 hours

The nature of local contagion may explain our findings. Local contagion brings information to a user through neighbors whom s/he trusts, whereas global contagion can be promoted by algorithms. Another explanation involves the nature of the datasets. DS2 contains tweets written in English. Thus, many users worldwide can understand and retweet as, for example, when external regimes interfere with U.S. politics [64] or in discussions about movies, TV shows, and sports [2]. The high engagement of users about different topics is expressed by short inter-retweet times and call for a deeper analysis of topic-contagion spread.

### 6.5 Contagion spread of a topic

This section aims to detect and measure for viral and non-viral topics the reach depth of local and global contagion. Each topic contagion event *W* in DS3 can contain several original tweets *w*_*μp*_ ∈ *W* about the Higgs boson particle.

**Viral events**. The percentage of global topic-contagion of viral events (16.9%) in DS3 is lower than the percentage of global contagion of viral original tweets (21.75%) in DS1 (Table 2). In terms of the contagion spreading distance from the tweeting source (*d*) of viral events (Fig 3e), DS3 presents similar trends to the contagion spread of original tweets in DS1 (Fig 3a), as summarized in Table 3. In DS3, for *d* ≤ 17, *d* ≠ 10, if a path exists on *G*, the more distant a user is from the user originating the information (source User *v*_0_), the less likely s/he is to retweet *w*_*μp*_ ∈ *W*.

Both DS1 and DS3 present a shift in trend for *d* = 10. In DS1, a shift is observed for *d* ∈ [10, 12], while in DS3 a shift is observed only for *d* = 10. This trend shift might be attributed to global contagion. Also, local topic-contagion spread of viral events (DS3), and local contagion spread of viral original tweets (DS1), are the most frequent (*d* = 1).

Considering the distance from the closest adopter (*d*_*ca*_ in Table 1), viral global topic-contagions in DS3 (Fig 3e), present similar trends (for *d*_*ca*_ ≤ 9) as viral global events in DS1 for *d*_*ca*_ ≤ 8 (Fig 3a). The larger *d*_*ca*_, the less likely a user is to retweet *w*_*μp*_ ∈ *W* in DS3 and, similarly, the less likely a user is to retweet an original tweet *w* in DS1. This trend is reversed for *d*_*ca*_ > 9 in DS3 and for *d*_*ca*_ > 8 in DS1.

**Non-viral events**. For non-viral events, the percentage of global topic-contagion (Table 2) in DS3 (12.8%) is similar to that in DS1 (12.14%), but smaller than in DS2 (18.29%). The trends of the contagion-spreading distances (*d*) for non-viral events in DS1 (Fig 3b), in DS2 (Fig 3d), and in DS3 (Fig 3f) are similar. For *d* ≤ 13 in DS3, similarly to DS1 and DS2 (Table 3), if a path exists on *G*, the more distant a user is from the source User *v*_0_, the less likely s/he is to retweet *w*_*μp*_ ∈ *W*. We also observe that local contagion spread of non-viral events in DS1, DS2, and DS3 are the most frequent ones (*d* = 1). Considering the distance from the closest adopter (*d*_*ca*_), in all three datasets (Fig 3b, 3d and 3f), global contagion spread presents similar trends. In DS1 and DS3 for *d*_*ca*_ ≤ 7, the closer a user to an adaptor, the more likely that user will retweet, while in DS2 the findings are similar for *d*_*ca*_ ≤ 6.

In all three datasets, local contagion is the most frequent contagion mechanism. The results show similar contagion spreading trends of (i) Viral information and viral topics, and (ii) Non-viral information and non-viral topics. In Fig 3, a contagion at *d* > 1 does not necessarily indicate node-to-node contagion spread and might be attributed to global contagion as well.

Based on the results of the data analysis in the present section, we test the three hypotheses of this study (Section 5).

## 7 Hypotheses testing

The results in the previous section pave the way to focusing in the present section on the findings of Hypothesis testing. Section 7.1 presents the testing of *H1*, based on the temporal spreading patterns of local and global contagion. Section 7.2 presents the testing of *H2*, using the findings about local and global contagion spread while, at the same time, considering the information virality by using the BIT approach developed in Section 3.4. Finally, Section 7.3 presents the testing of *H3* regarding the similarity between contagion spread patterns of a topic to those of particular tweets.

### 7.1 *H1* testing

To test *H1* and reveal whether local and global contagion has different temporal spreading patterns, we compare the distribution of the ECDFs in Fig 4 above by using the Kolmogorov-Smirnov (KS) D-statistic test [18, 65]. The D-statistic is defined as the maximum distance: *D* = *max*(|*F*_1_(*x*) − *F*_2_(*x*)|), where *x* represents the range of the random variable, and *F*1 and *F*2 represent the empirical cumulative distributions functions. The smaller the distance, the more similar the distribution curves and, hence, the more likely are the two samples to come from the same distribution. In the KS-test, a *p*_*value*_ < 0.05 indicates that the samples are not drawn from the same distribution.

For DS1, we found support for *H1* via four KS-tests for pairs of ECDFs (Fig 4a) and found, as elaborated upon in Table 6, significant differences between the ECDFs (with varying D-statistics and every *P*_*value*_ ≤ 2.2 × 10^−16^). Thus, we found the temporal spreading patterns of local and global contagion to be significantly different. Also, for DS2, similar to DS1, we found support for *H1* via a single KS-test between the ECDFs of local and global contagions (Fig 4b). The KS-test revealed that the two ECDFs are significantly different (D-statistic 0.56, *P*_*value*_ ≤ 2.2 × 10^−16^), thus uncovering different patterns of user behavior. In sum, the KS-tests for both DS1 and DS2 support *H1* by revealing that local and global contagion have significantly different temporal spreading patterns.

**Table 6:** Kolmogorov-Smirnov results.

DS	#	Pairs of ECDFs tested by a KS-test	D-statistic	Figure
DS1	1	Non-viral local, Viral local	0.48*	Fig 4a
	2	Non-viral global, Viral global	0.55*	
	3	Non-viral global, Non-viral local	0.16*	
	4	Viral global, Viral local	0.07*	
BIT DS1	1	Non-viral local, BIT local	0.58*	Fig 4c
	2	Non-viral global, BIT global	0.51*	
	3	Non-viral global, Non-viral local	0.16*	
	4	BIT global, BIT local	0.13*	
DS3	1	Non-viral local, Viral local	0.37**	Fig 4d
	2	Non-viral global, Viral global	0.47**	
	3	Non-viral global, Non-viral local	0.12**	
	4	Viral global, Viral local	0.09**	

*P_value_ < 2.2 × 10^-16^;

**P_value_ < 2.2 × 10^-22^

Aiming to go beyond examining and explaining local versus global contagions, we apply next, the Back-in-time (BIT) approach for early detection of viral posts before becoming viral.

### 7.2 *H2* testing

Next, to test *H2* and reveal if viral and non-viral information have different local and global contagions spreading patterns, we use the BIT approach for DS1 since DS1 contains both viral information, needed for applying the BIT approach, and non-viral information.

**Fig 5:**
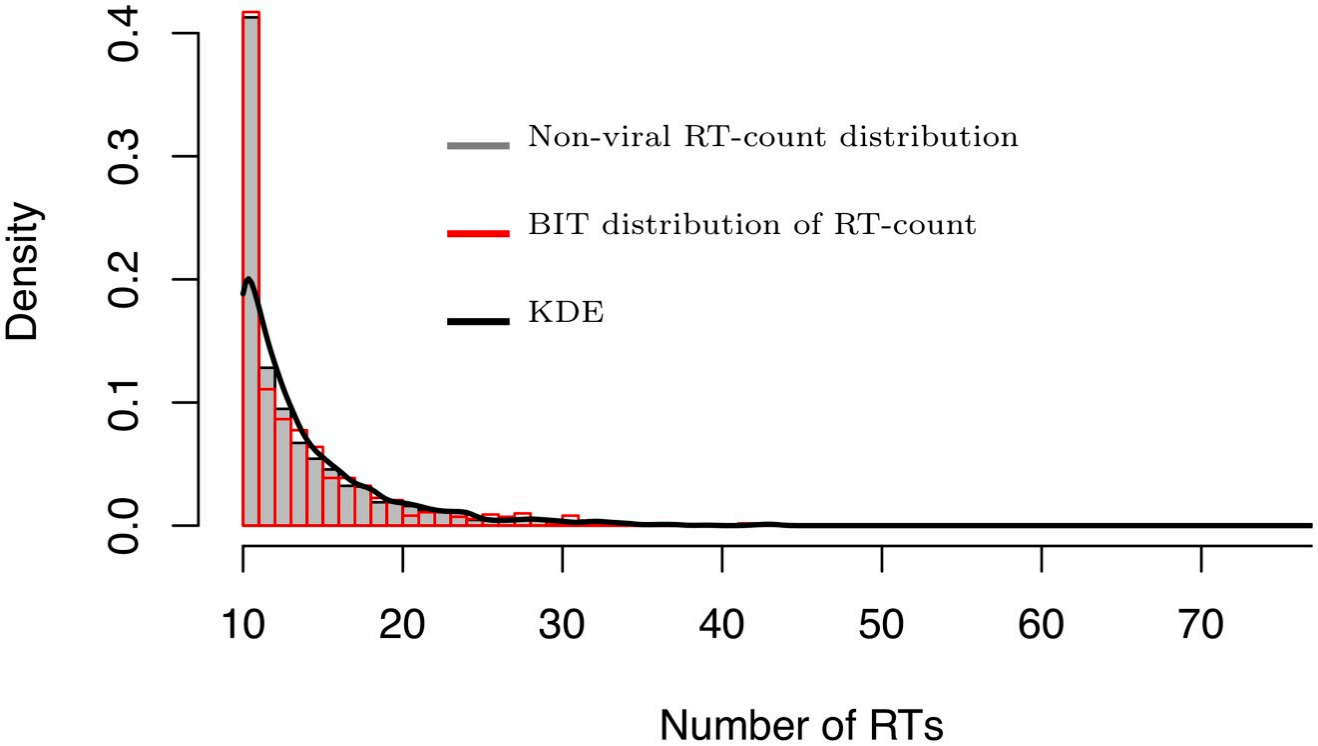
Retweet-count distribution of BIT and non-viral tweets.

Applying the developed BIT approach to DS1 in Step *i*, we created a KDE to estimate the probability density function of the retweet-count based on 1,950 non-viral tweets (Table 2). Having found in Step *ii* (Table 2) that the number of original viral tweets was 127, we sampled therefore 127 numbers from the KDE and randomly assigned a BIT retweet-count *y* to each of the 127 viral tweets. In Step *iii*, we rolled back in time each of the 127 viral tweets to a point where its retweet-count was *y*. We repeated Steps *ii* to *iii* 100 times. Fig 5 presents for DS1 the retweet-count distribution of non-viral information, the KDE, and the distribution of the KDE samples, i.e. the distribution of the retweet-count of BIT tweets obtained in Step *ii*.

The BIT-tweets are viral tweets that were rolled back in time to a point when their retweet-count was 10 to 99, the same as the retweet-count for non-viral tweets. We then assigned to each BIT tweet a contagion type. Fig 4c above presents two local and global curves for the BIT tweets and two local and global curves for the non-viral tweets. As depicted in Fig 4c, although the BIT tweets have a smaller retweet count compared to viral tweets, their contagion spread patterns are similar to those of viral tweets and the ECDFs of BIT tweets are located above the ECDFs of non-viral tweets. In addition, local contagion is located above global contagion for both non-viral tweets and BIT tweets.

Support for *H2* was found via four KS-tests by analyzing the four curves in Fig 4c based on DS1 for: local non-viral versus local BIT tweets, global non-viral versus global BIT tweets, global non-viral tweets versus non-viral local tweets, and global BIT tweets versus local BIT tweets. As elaborated upon in Table 6, all ECDFs are significantly different from one another (with varying D-statistics and every *P*_*value*_ ≤ 2.2 × 10^−16^). Finding support for *H2* suggests that both the global and the local contagion spreading patterns of the BIT tweets significantly differ from the patterns of non-viral tweets.

### 7.3 *H3* testing

Based on DS1 and DS2 analyses, *H1* and *H2* testing focused on contagion spread of a single original tweet. However, contagion of an idea or a topic is not limited to a single tweet and can spread by a sequence of tweets discussing the same topic, as in the DS3 dataset, which is devoted to the announcement about the discovery of the Higgs boson particle.

To test *H3* and reveal if topic contagion has similar temporal spreading patterns to information contagion spread, we studied the inter-retweet time of topic contagion spread. Fig 4d presents four ECDFs of inter-retweet times that correspond to each contagion type—local versus global and each event virality—viral versus non-viral. Similar to the inter-retweet patterns of viral original tweets in DS1 (Fig 4a), Fig 4d shows for DS3 that the two ECDFs for local and global viral events are located above the respective ECDFs for non-viral events. Similar to the inter-retweet patterns of non-viral original tweets in DS1 (Fig 4a), Fig 4d also shows for DS3 that the global contagion curves are located below the local contagion curves, up to point *P*_2_. After *P*_2_, the global contagion curves slightly exceed the local contagion curves. In DS3, user interaction occurs before, during, and after the announcement of the Higgs boson discovery and different time-varying dynamics of user activities were found in these three periods [53]. The popular media’s coverage of the events toward the last period of the data collection, led most likely to global contagion [53]. In both DS3 and DS2, as depicted in in Fig 4d and 4b respectively, the ECDFs for local and global contagion of non-viral events alternate their relative location at *P*_1_ and *P*_2_. This alternation implies for non-viral events the sensitivity of global contagion spread to exposure by global contagion sources like the mainstream media. Like in DS1 and as elaborated in Table 6 for DS3, we found support for *H3* by conducting four KS-tests. The results revealed that the four ECDFs curves are significantly different (with varying D-statistics and every *P*_*value*_ ≤ 2.2 × 10^−22^).

## 8 Conclusions

Most contagion research is bound by modeling local contagion and the spread of viral information only. This study analyzed and compared user behavior in three Twitter datasets and found, contrary to the common assumption that contagion diffuses from node-to-node, that contagion in OSNs also spreads globally beyond social network links. Our finding of significant differences in the spread of global versus local contagion of viral and non-viral information implies different mechanisms of contagion. We also found that the contagion spread of a topic presents similar patterns to the contagion spread of a single information nugget (original tweet). Regarding the spread of global contagion of non-viral events (original tweets or topics), we found that the larger the distance of a user from the closest adopter, the less likely s/he is to be globally infected. For all three datasets, this trend is reversed approximately at a distance of 7 from the closest adopter and at a distance of 8 from the closest adopter for global contagion of viral events. These findings can be explained by user reluctance to search for information via user-to-user page crawling at these distances. Thus, contagion more likely occurs due to content promoted by external sources that facilitate jumps between content pages, like content recommendation algorithms, mass media, or searching for information in the Twitter search box.

We also analyzed the temporal retweeting activity of users and found that viral information spreads faster than non-viral information. In addition, the patterns of local and global contagion of non-viral information depend on the nature of the analyzed dataset, probably since retweeting non-viral content, is less likely covered by mainstream media and is less appealing. Therefore, contagion spread depends on content promoting algorithms like Twitter Timeline.

KS-tests revealed that local and global contagion have significantly different spreading patterns, supporting *H1*. Analysis of inter-retweet times revealed significant differences between the spreading patterns of viral and back-in-time tweets, supporting *H2*. In support of *H3*, we found that the contagion spreading patterns of viral and non-viral topic contagion events are similar to the contagion spreading patterns of viral and non-viral information. Other than the contributions of this work via hypothesis testing, another innovative contribution is the development of the back-in-time (BIT) approach used to analyze the spreading patterns of viral tweets at a point back in time when they had a retweet-count of non-viral tweets.

One limitation of this study is that the size of the Twitter Following-list might have changed during the data-collection period, possibly influencing user exposure to information. To test the change in size of a user’s Following-list, we recollected two weeks after the initial collection date the Following-lists of randomly selected 538 users from DS2. Our finding that 80% of users had an absolute difference between their Following-lists of less than 53 reveals that most users had a minor change. Another limitation is that some tweets can become viral after the collection date. Therefore, non-viral tweets were queried using Twitter API at least a month after the date they were posted, verifying that their retweet counts did not grow to be viral.

Whereas most studies focus on user-to-user contagion, our focus is on how viral and non-viral contagion spread. Since most content is non-viral, its analysis makes a valuable contribution to the OSN research. The novelty of this study is in modeling user behavior in an OSN, accounting for local and global effects, and in developing the BIT approach.
